# Early-life maternal attachment and risky health behaviours in adolescence: findings from the United Kingdom Millennium Cohort Study

**DOI:** 10.1186/s12889-021-12141-5

**Published:** 2021-11-08

**Authors:** Beatrice D. Reyes, Dougal S. Hargreaves, Hanna Creese

**Affiliations:** Department of Primary Care and Public Health, School of Public Health, Imperial College London, W6 8RP, London, UK

**Keywords:** Maternal attachment, Multiple risky behaviours, Alcohol consumption, Gambling, Antisocial behaviour, Criminal engagement, Smoking, Vaping, Unsafe sex, Illegal drugs

## Abstract

**Background:**

Early uptake of multiple risky behaviours during adolescence, such as substance use, antisocial and sexual behaviours, can lead to poor health outcomes without timely interventions. This study investigated how early-life maternal attachment, or emotional bonds between mothers and infants, influenced later risky behaviours in adolescence alongside other potential explanatory pathways using the United Kingdom Millennium Cohort Study.

**Methods:**

Total maternal attachment scores measured at 9 months using the Condon (1998) Maternal Postnatal Attachment Scale compared higher and lower attachment, where mothers in the lowest 10th percentile represented lower attachment. Multiple risky behaviours, defined as two or more risky behaviours (including smoking cigarettes, vaping, alcohol consumption, illegal drug use, antisocial behaviour, criminal engagement, unsafe sex, and gambling), were scored from 0 to 8 at age 17. Five multivariate logistic regression models examined associations between maternal attachment and multiple risky behaviours among Millennium Cohort Study members (*n* = 7796). Mediation analysis sequentially adjusted for blocks of explanatory mechanisms, including low attachment mechanisms (multiple births, infant prematurity, sex, breastfeeding, unplanned pregnancy and maternal age at birth), maternal depression, and social inequalities (single-parent status, socioeconomic circumstance by maternal education and household income) at 9 months and poor adolescent mental health at 14 years.

**Results:**

Children of mothers with lower maternal attachment at 9 months had 23% increased odds of multiple risky behaviours at 17 years (OR: 1.23, 95% CI: 1.00–1.50) in the unadjusted baseline model. All five explanatory blocks attenuated baseline odds. Low attachment mechanisms attenuated 13%, social inequalities 17%, and poor mental health 17%. Maternal depression attenuated the highest proportion (26%) after fully adjusting for all factors (30%).

**Conclusions:**

Lower maternal attachment in early life predicted increased adolescent multiple risky behaviours. Almost a third of the excess risk was attributable to child, maternal and socioeconomic factors, with over a quarter explained by maternal depression. Recognising the influence of early-life risk factors on adolescent health could innovate current policies and interventions addressing multiple risky behaviour uptake affecting health inequalities across the life course.

**Supplementary Information:**

The online version contains supplementary material available at 10.1186/s12889-021-12141-5.

## Background

Preventing modifiable risky health behaviours adopted during adolescence is key to reducing population health inequalities. In recent years, the life course approach has provided a holistic and interdisciplinary perspective informing public health priorities, proposing that differential exposures accumulated during “critical” life stages have lasting implications on health outcomes [1, 2]. However, the influence of early-life dynamics on adolescent outcomes are not yet fully understood. In 2017, the World Health Organisation highlighted the role of childhood psychosocial determinants, such as stress, resilience and social relationships, which are significant yet overlooked factors neglected in policy and intervention frameworks [2]. As a result, understanding how children learn to manage stress from an early age through caregiver-child relationships, namely emotional bonds formed through attachment, may serve as a potential explanation for underlying population health inequality due to risky health behaviours [3].

Few studies have examined associations between early-life maternal attachment (MA) and multiple risky behaviours (MRBs), and underlying mechanisms remain unclear. Current analyses are typically limited to cross-sectional studies, lack adjustment for MA or studied risky behaviours in isolation, consequently preventing inquiry into key explanatory variables across childhood [4–7]. A systematic review by Meader et al. (2016) revealed that risky behaviours, namely risky sexual behaviour, alcohol, illegal drug and smoking consumption, typically cluster and co-occur in young adults [8]. Therefore, targeting multiple harms may be more efficient than interventions focusing on individual health behaviours [8]. At present, only two meta-analyses have investigated the importance of attachment and risky behaviours in isolation. One paper by Kim and Miller (2020) analysed lower, or “insecure”, attachment styles and unsafe sexual behaviour, while another by Fairbairn et al. (2018) focused on substance use [9, 10]. Both analyses demonstrated significant associations between lower attachment and poor health outcomes due to risky behaviours. Of note, Fairbairn et al. concluded that attachment in early childhood predicted later substance use, emphasising that further inquiry into early-life attachment pathways were necessary for future research [10].

Previous systematic reviews highlight several significant risk factors consistently associated with MA and MRBs, which may act as mechanisms influencing child health and development. For example, children affected by low socioeconomic circumstances (SECs), including single-parent households, less-educated mothers and lower household incomes, may lack higher MA due to longer working hours [11]. SECs affected by maternal education are also indicative of maternal health literacy and health behaviours, which children may model [12, 13]. Likewise, risk factors associated with low MA, such as a lack of breastfeeding, multiple births, unplanned pregnancy, infant prematurity and young maternal age, may confound increased MRB uptake [14, 15]. Mothers with depressive symptoms are also at higher risk of developing low MA with infants, while adolescents with poor mental health may adopt MRBs to cope with stress [16, 17]. As a result, there is a need to conceptualise these mechanisms through longitudinal research to inform timely evidence-based interventions.

The primary aim of this study was to test whether MA formed during infancy was associated with subsequent risky health behaviours during adolescence. We use United Kingdom (UK)-representative data, the Millennium Cohort Study (MCS), to investigate whether low attachment mechanisms, poor mental health in mothers at 9 months and adolescents at 14 and social inequalities were potential underlying explanatory factors affecting the association between MA and MRBs.

## Methods

### Data

Using data from the MCS, we investigated associations between early-life MA at 9 months and adolescent MRBs at 17 years old in the UK. The MCS is a nationally representative prospective cohort study following the lives of 18,296 singleton children born from September 2000 to January 2002 [18]. A total of seven data collection sweeps were conducted at 9 months, 3, 5, 7, 11, 14 and 17 years old. Responses were received from cohort members and parents, where mothers were typically the main parent respondent. Oversampling participants from ethnic minorities and disadvantaged socioeconomic backgrounds was conducted to ensure sufficient statistical power in these smaller subgroups. Stratified clustered sampling was employed to broadly represent all four countries in the UK (England, Wales, Scotland and Northern Ireland) [19]. Study weightings were applied to account for the sampling design and adjust for non-response at each cross-sectional sweep. More information on the sampling methods and the cohort profile is detailed online (https://cls.ucl.ac.uk/cls-studies/millennium-cohort-study/). MCS datasets were accessed and downloaded from the UK Data Service website, and all analyses were performed on Stata/IC 16.0 [20].

### Ethical approval

The MCS secured ethical approval from the National Health Service Multi-Centre Research Ethics Committee (NHS MREC) for each sweep. All methods comply with the relevant national and institutional committees on human experimentation, including the ethical standards outlined in the 1975 Helsinki Declaration, as revised in 2013 [21, 22]. With cohort members under 18, written informed consent was required and obtained from parents/legal guardians. Following interviews, ongoing support was also made available to cohort members and parents through information booklets, where links to professional support services and helplines were provided [23]. This study required no further ethical approval.

### Inclusion and exclusion criteria

**Fig. 1:**
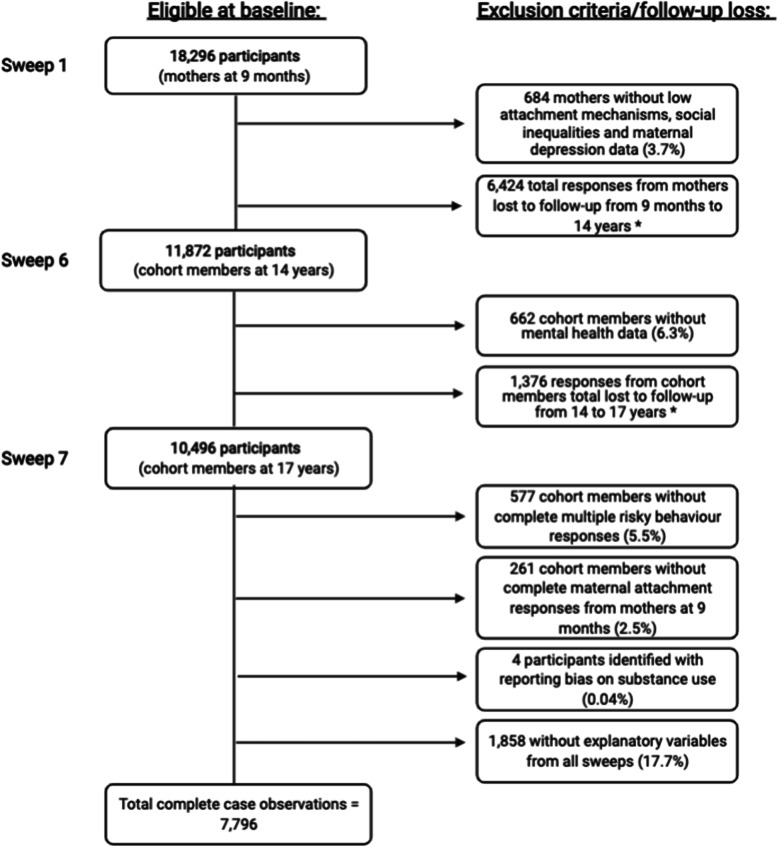
Total participants following study exclusion and follow-up loss at each studied sweep. Legend/note: Potential explanatory variables at 9 months include infant sex, low attachment mechanisms (breastfeeding status, unplanned pregnancy, infant prematurity, maternal age at birth, multiple births), maternal depression, social inequalities (socioeconomic circumstance by maternal education, household income by UK quintiles, single-parent status) and poor adolescent mental health measured at 14 years. Asterisks (*) indicate loss to follow-up, which was accounted for by attrition weightings

The first data collection sweep at age 9 months included 18,296 singleton-born children. However, by the seventh sweep, participating families had declined to 10,496 due to attrition commonly affecting cohort studies (Fig. 1). The MCS has weightings for attrition to account for follow-up loss from previous cross-sectional sweeps of surveys [24]. Nonetheless, the overall response rate was an acceptable 74% at 17 years, suggesting that missingness was largely due to follow-up loss rather than questionnaire response [23]. Additional comparison between the omitted and analytical complete case samples was conducted and presented in Additional file 1 – List of Appendices: Appendix Table 1. No significant differences were observed, so a Missing Completely At Random assumption was undertaken.

### Exposure, outcomes and potential explanatory variables

#### Exposure

Emotional ties experienced by mothers to infants, or MA, can characterise how sensitive caregivers are to meeting infant needs [25]. Other terms such as “mother-to-infant bonding” or “mother-to-infant attachment” are also used in the literature to describe this relationship [26]. Related to this concept is “attachment”, where infants have an innate biological need to seek parental caregiving responses and reassurance in stressful situations, first described by Bowlby in 1969 [27]. Before children independently manage distress, mothers regulate infants’ distressful emotions during early childhood according to different maternal sensitivities. Attachment may influence how children form later relationships with others, manage emotions and health behaviours [3]. As a result, such early childhood dynamics influenced by MA are significant to nurturing the foundations of healthy child development.

Mothers’ MA with infants was measured at 9 months using mean scores from a modified 6-item Maternal Postnatal Attachment Scale originally developed by Condon (1998) [28]. Mothers self-reported thoughts and feelings towards their infant from a selection of options provided (see Appendix Table 2 for MA questions and responses). Mean scores ranged from 1 to 6, with higher scores indicating better attachment. We followed methods by Wadman et al. (2020), where total attachment scores were dichotomised according to the lowest 10% (mean scores below and equal to 3.5) of the sample to represent “lower” attachment, while the remaining 90% were “higher” [29]. To better conceptualise MA, it was kept as a binary measure. MA was additionally studied as a continuous variable in Appendix Table 3. The internal reliability of the modified questionnaire using mean scores had a Cronbach’s alpha coefficient (α) of 0.82 [30].

#### Outcome

Risky health behaviours among cohort members at 17 were surveyed in the MCS in the seventh sweep. The questionnaire investigated a total of 8 behaviours occurring within the last 12 months: smoking cigarettes, vaping, illegal drug use, alcohol consumption and binge-drinking, antisocial behaviour, gambling, criminal engagement, and unsafe sex (see Appendix Table 4 for MRBs questions and response options). All questions were self-reported by cohort members using a tablet provided by the interviewer [24].

##### Alcohol consumption, smoking and vaping

Most questions required binary “yes” or “no” responses, apart from alcohol, smoking and vaping variables which were measured according to frequency. We compared adolescents reporting regular alcohol consumption (1–2, 3–5, 6–9, 10–19, 20–39 or 40 or more times) and binge drinking (“>5 alcoholic drinks in one sitting”) against those who had never tried alcohol. Regular cigarette and vape smokers were also defined as cohort members who reported current use (“sometimes but not as often as once a week” to “more than six times a week”), while non-regular smokers were those with previous attempts i.e. “tried once”, “sometimes but never smoke now” to “never” over the past year.

##### Drug use

Questions on whether adolescents had tried marijuana, cocaine, acid/LSD, ecstasy, heroin, crack, speed/amphetamines, methamphetamine, Semeron (a fictional drug), ketamine, mephedrone or psychoactive substances, such as mushrooms or salvia, were included.

##### Gambling

Questions asked cohort members whether they had experiences with gambling on fruit machines, betting with friends, private betting or any other format.

##### Unsafe sex

Whether participants had sex without contraception, failed to test for STIs, pregnancies, or a positive diagnosis following an STI test were considered risky sexual behaviour.

##### Antisocial behaviour

Antisocial behaviour topics ranged from whether they had: been a public nuisance (“noisy or rude in a public space”), graffitied, vandalised, shoplifted, stolen from someone (e.g. mobile phone, money), burgled someone’s home, assaulted (shoved, hit, slapped, punched someone), assaulted someone with a weapon, carried a knife or weapon, part of a gang, hacked or sent a virus (cybercrime), stolen a vehicle, deliberately set something on fire that should not have, harassed someone (including sexual harassment), used someone else’s credit card, sent pictures or spread rumours about someone via email or phone.

##### Criminal engagement

Questions on whether they had ever been stopped and questioned, cautioned or warned, or arrested by the police were asked.

A sum score of risky behaviours was created by adding all eight individual risky behaviours together, with scores ranging from 0 (no uptake) to 8 (all risky behaviours). MRBs were dichotomised to compare MA differences between one or none against 2 or more risky behaviours following the MRBs definition by Hale and Viner (2016) [6].

#### Potential explanatory variables

Following a literature review, we divided significant potential explanatory factors for MA and MRBs into low attachment mechanisms, infant sex, social inequalities and poor mental health in mothers and adolescents. A logic model illustrating the associations between MA, MRBs and potential explanatory factors is presented in Appendix Fig. 1 [31]. Confounders are variables associated with both exposure and outcome measures but are not on the causal pathway between them [32]. Mediators are hypothesised to be on the causal pathway, occurring after the exposure and before the outcome. Effect modifiers, also known as moderators, are not on the causal pathway but may modify the direction or size of the association for different groups in the analysis (e.g. comparing boys and girls). By contrast, covariates only affect the exposure or outcome measure.

An explanation of how these variables were measured are detailed below.

##### Low attachment mechanisms

Factors that may increase the risk of low attachment include breastfeeding status (< 3 months compared to > 3 months), unplanned pregnancy, infant prematurity (indicated by < 37 gestation weeks), multiple births and maternal age at birth.

##### Social inequalities

SECs by maternal education (National Vocational Qualification/NVQ levels), household income by UK quintiles and single-parent status were all measured at 9 months.

The MCS combined academic and professional measures according to NVQ levels on a scale of 1 to 5 [33]. NVQ Level 1 corresponds to General Certificate of Secondary Education (GCSE) grades D-G, while grades A*-C are in NVQ Level 2. NVQ Level 3 includes Advanced (A)-levels, which precedes levels 4 and 5 equating to bachelor’s degrees and higher degrees respectively. More information on UK education qualifications and their international equivalents can be found on the UK government website (https://www.gov.uk/what-different-qualification-levels-mean/list-of-qualification-levels).

Household incomes were equivalised into quintiles by the MCS through a modified version of the OECD (Organisation for Economic Co-operation and Development) scales. Family needs were compared to a baseline of a couple with no children at 1. More information on how the MCS measured household quintiles is detailed in the MCS User Guide under the “Equivalisation” section (http://doc.ukdataservice.ac.uk/doc/5795/mrdoc/pdf/mcs1-5_user_guide_ed9_2020-08-07.pdf).

##### Maternal mental health

Maternal depression was assessed using a selection of 9 items from the Rutter Malaise Inventory at 9 months, which showed adequate reliability of at least α = 0.70 [34, 35]. Selected by John Brynner, these items asked mothers to indicate feelings of tiredness, depression, worry, rage, fear, upset and nervousness (including constant jitters and heart-racing) through yes or no statements [35]. Total maternal depression scores were added together and kept as a binary yes or no measure. Higher scores indicated increased depressive symptoms among mothers.

##### Adolescent mental health

A total of 13 questions were asked on adolescent mental health at 14 years using a shortened version of the Moods and Feelings Questionnaire first created by Angold et al. (1995) [36]. Cohort members were asked to report whether they recently felt: “miserable or unhappy”, “lonely”, “didn’t enjoy anything at all”, “felt so tired that they sat around and did nothing”, “restless”, felt they were “no good anymore”, “cried a lot”, found it “hard to think properly or concentrate”, “hated themselves”, felt that they were a “bad person”, thought that “nobody really loved them”, thought that they could “never be as good as the other kids” or “did everything wrong” over the last 2 weeks (α = 0.93). A sum score for poor adolescent mental health distinguished between non-depressed and depressed adolescents using a cut-off score of > 12 to indicate clinically relevant depressive symptoms following methods by Thabrew et al. (2018) [37].

### Statistical analysis

We undertook a complete case analysis following an acceptable response rate of 74% and a comparison between omitted and analytical samples (Appendix Table 1) [23]. Attrition weights were applied at each studied sweep to account for missing data due to follow-up loss up to the seventh sweep at age 17.

We first estimated the prevalence of risky behaviours among cohort members at 17 years by MA at 9 months. Bivariate associations between risky health behaviours, such as smoking and vaping, were examined. These logistic regressions in Appendix Table 5 assessed the co-occurrence of risky behaviours and that a sum score of MRBs was suitable for this study. MA was studied as a binary variable following methods by Wadman et al. (2020), yet associations as a continuous variable are also presented in Appendix Table 3 [29].

Guided by the logic model (Appendix Fig. 1), we used multivariate logistic regression analysis to compare MRB uptake among adolescents at 17 by higher or lower MA scores at 9 months. The baseline model first assessed univariate associations between MA and MRBs. Adjusting for blocks of potential explanatory factors in a stepwise manner, we then examined how the odds ratio (OR) changed on the inclusion of each block of potential explanatory factors in five models through mediation analysis.

Model 1 adjusted for low attachment mechanisms (multiple births, unplanned pregnancy, infant prematurity, breastfeeding and maternal age at birth), Model 2 adjusted for maternal depression, Model 3 adjusted for poor adolescent mental health, Model 4 adjusted for socioeconomic factors (single-parent status, SECs by maternal education and household income by UK quintiles) and Model 5 fully adjusted for all explanatory factors. All models except the baseline adjusted for infant sex. To estimate the change in OR of MRBs for those with lower compared to higher attachment, we calculated the difference as 100 x (baseline OR – adjusted OR) / (OR – 1), whereby adjusted ORs accounted for blocks of explanatory factors [38, 39].

Causal pathways are challenging to understand. Explanatory factors are expected to cluster and are difficult to interpret in isolation. We, therefore, took a sequential “en-block” approach through mediation analysis to examine the cumulative effect of blocks of potential mediating and confounding factors, considering low attachment mechanisms, maternal mental health, adolescent mental health, socioeconomic factors [40]. Pearson goodness-of-fit tests were applied after logistic regression models to assess whether there was no departure from logistic regression assumptions and no interactions were present, provided that the resulting *p*-value was non-significant (> 0.05). Multi-collinearity, variables explaining the same variation in a model, was also examined through mean Variance Inflation Factors. All six logistic regression models (including the baseline) in this study met their required assumptions. Analyses were conducted on Stata/IC 16.0.

### Sensitivity analysis

As alcohol consumption tends to be patterned differently than other risky behaviours, with adolescents from high-income backgrounds more likely to consume alcohol than low-income peers, we conducted sensitivity analyses of the association between MA and MRBs without alcohol consumption [41].

## Results

### Descriptive results

A total of 7796 children had complete case observations on exposure, outcome and potential explanatory variables from the first (9 months), sixth (14 years) and seventh (17 years) sweeps (Fig. 1). No significant differences between omitted and complete case samples (see Appendix Table 1) were observed following an acceptable survey response rate of 74% before employing a Missing Completely at Random assumption [23].

At 9 months, 6907 mothers had higher MA (88.72, 95% Confidence Intervals (95% CI): 87.73–89.64%), while 889 had lower attachment (11.28, 95% CI: 10.36–12.27%). Table 1 shows that 2588 cohort members (31.43, 95% CI: 29.84–33.06%) reported no MRB uptake, while 5208 (68.57, 95% CI: 66.94–70.16%) had engaged in 2 or more risky activities at 17 years old. Children of mothers who reported lower attachment were more likely to engage in MRBs at 17 years old (72.36, 95% CI: 68.13–76.22%) than mothers reporting higher attachment (68.09, 95% CI: 66.43–69.70%).

**Table 1|:** Estimated risky behaviour prevalence at 17 years by mothers’ MA at 9 months

Risky behaviour	Maternal attachment					
	Higher		Lower		Total	
	^b^ n	^c^ %	n	%	n	%
^a^ MRBs at 17	4611	68.1	597	72.4	5208	68.6
Regular smoking	1371	21.8	186	23.6	1557	22.0
Regular vaping	784	11.7	110	12.7	894	11.8
Alcohol consumption and binge drinking						
Yes – but never binged	1881	28.5	234	26.6	2115	28.4
Yes – and have binged before	3754	58.5	467	58.6	4221	58.4
Illegal drugs	2004	31.1	302	37.6	2306	31.9
Gambling	691	14.0	85	11.7	777	13.7
Criminal engagement	937	18.0	136	18.8	1073	18.1
Antisocial behaviour	2129	31.0	283	35.7	2412	31.6
Unsafe sex	2589	40.0	328	42.1	2917	40.3

**Footnote:** MA = maternal attachment. ^a^ MRBs = Multiple Risky Behaviours, ^b^ n = number of observations, ^**c**^ % column percentage. MRBs in cohort members at 17 years include smoking, vaping, alcohol consumption/binging, illegal drugs, gambling, criminal engagement, antisocial behaviour and unsafe sex. Total number of complete case sample participants (n) = 7796

With the exception of alcohol consumption, all individual risky behaviours had increased odds of partaking in other risky behaviours (Appendix Table 5). For example, adolescents who regularly smoked had 12.7 times the odds of regularly vaping (OR: 12.70, 95% CI: 10.55–15.48). The highest association between risky behaviours was between regular smoking and illegal drugs (OR: 13.22, 95% CI: 11.20–15.61).

Adolescent alcohol consumption was associated with decreased odds of regular vaping (OR: 0.87, 95% CI: 0.57–1.33) and gambling (OR: 0.86, 95% CI: 0.61–1.22).

### Multivariate regression results

Lower attachment at 9 months among mothers was associated with a higher OR of MRBs at 17 years in the unadjusted baseline model (OR: 1.23, 95% CI: 1.00–1.50). Table 2 shows the extent to which the elevated OR of MRBs by attachment during early life was attenuated when adjusting for blocks of potential explanatory factors in a stepwise manner.

**Table 2|:** Odds ratios of MRBs by lower MA adjusted for explanatory models

Model	Multiple risky behaviours by lower maternal attachment		Proportion attenuated (%)
	OR	95% CI	
Baseline	1.23*	1.00–1.50*	Reference
1: Low attachment mechanisms	1.20	0.98–1.47	13%
2: Maternal depression	1.17	0.95–1.44	26%
3: Poor adolescent mental health	1.19	0.97–1.45	17%
4: Social inequalities	1.19	0.98–1.45	17%
5: Fully adjusted	1.16	0.95–1.41	30%

**Footnote:** * = *p* < .05, OR = Odds Ratio, 95% CI = 95% Confidence Intervals. Baseline model = unadjusted univariate analysis between MA (maternal attachment) and MRBs (multiple risky behaviours); Model 1 (low attachment mechanisms at 9 months) = adjusted for multiple births + unplanned pregnancy + infant prematurity + breastfeeding + maternal age at birth + infant sex; Model 2 (maternal depression) = adjusted for maternal depression at 9 months + infant sex; Model 3 (poor adolescent mental health) = adjusted for poor adolescent mental health at 14 + infant sex; Model 4 (social inequalities at 9 months) = adjusted for single parent status + socioeconomic circumstance by maternal education + household income + infant sex and Model 5 was fully adjusted for all risk factors. Proportion attenuated calculated by the formula: (100 x (Baseline model OR – Adjusted OR) / (Baseline model OR – 1)). Total number of complete case observations was 7796. Sensitivity analysis results presented in Additional file 1 – List of Appendices, Appendix Table 6

Adjustment for low attachment mechanisms (Model 1: multiple siblings, prematurity, breastfeeding, maternal age at birth, unexpected pregnancy and infant sex) led to a 13% attenuation in the OR of MRBs in adolescents from mothers with lower attachment (OR: 1.20, 95% CI: 0.98–1.47). Adjustment for maternal depression (Model 2) led to a 26% attenuation in the OR of MRBs in adolescents from mothers with lower attachment (OR: 1.17, 95% CI: 0.95–1.44). Adjusting for adolescent mental health (Model 3) saw a 17% attenuation in the baseline OR (OR: 1.19, 95% CI: 0.97–1.45). Adjustment for social inequality factors (Model 4, including single-parent status, household income by UK quintiles, maternal education according to NVQ levels) led to a 17% attenuation in the odds ratio of MRBs (OR: 1.19, 95% CI: 0.97–1.45). In the final model, adjusting for all blocks of explanatory factors together, the overall reduction in OR was 30%.

Sensitivity analyses findings without alcohol consumption were consistent with the associations observed by the main models in Table 2 (Appendix Table 6).

## Discussion

### Main findings and study implications

Having lower MA at 9 months increased the likelihood of partaking in MRBs in adolescence by 23%. Almost a third (30%) of the excess risk was attributable to child, maternal and socioeconomic characteristics during the early years of life, with the leading explanatory factor, maternal depression, accounting for 26% of the association between MA and MRBs.

### Comparison with existing literature

The majority of individual risky behaviours were significantly associated with each other, supporting recommendations from Hale and Viner (2016) and a systematic review by Meader et al. (2016) to target MRBs concurrently [6, 8]. Whitaker et al. (2021) found that substance use such as alcohol consumption, smoking and illegal drugs clustered in unhealthy adolescents, while risky sexual activity and gambling typically occurred in isolation [42]. By contrast, this study finds reduced odds in alcohol consumption with vaping and gambling, suggesting that adolescents who drink may not engage in other risky behaviours. A possible explanation from Hale and Viner (2016) cites the problem behaviour theory by Jessor et al. (1998), hypothesising that alcohol consumption is perceived as socially acceptable among adolescents and is adopted independently without other MRBs [6, 43].

Sensitivity analysis findings without alcohol consumption were consistent with previous research, suggesting that adolescents from wealthier backgrounds had a higher prevalence of drinking than poorer households [41]. An explanation from Collins (2016) proposes that higher disposable income and financial support from wealthier parents may enable adolescents to spend more money on alcohol than their socioeconomically disadvantaged peers [44]. As a result, taking perspectives from both Jessor et al. (1998) and Collins (2016), increased alcohol consumption among adolescents from affluent families may be due to a combination of higher financial opportunity and social acceptability [41, 43].

By contrast, other risky behaviours in this study were associated with low SECs, showing a reverse trend compared to alcohol consumption. Similar findings by Delker et al. (2018) from the United States National Institute of Child Health Development study show that insecure-disorganised children at 15 months with poor SECs were five times more likely to adopt MRBs at 15 years than securely attached children from affluent backgrounds (*n* = 1364) [45]. This study further reinforces social inequalities as a confounder between MA and MRBs and comparatively investigates a longer timeframe using a larger UK cohort. However, Delker et al. studied attachment from the child’s perspective, while this study focuses on the mothers’ feelings and thoughts. Uniting both perspectives could provide further insight into the influence of attachment or emotional ties between mothers and children on later health outcomes. Another UK study by Kipping et al. (2015) using the Avon Longitudinal Study of Parents and Children also found that 6406 adolescents aged 15–16 were more likely to engage in MRBs with each incremental decrease in SEC (OR: 1.22, 95% CI: 1.15–1.29), maternal education (OR: 1.15, 95% CI: 1.09–1.21) and income (OR: 1.12, 95% CI: 1.08–1.16) [46]. Such social patterning of risky behaviours associated with low SECs is also characteristic of a lack of health literacy and access to health facilities in deprived neighbourhoods, potentially contributing to increased MRB uptake among adolescents from lower-income families [46]. A systematic review by Reiss (2013) further emphasises the influence of socioeconomic inequalities on child mental health, where low SECs was strongly associated with higher mental health problems [47]. As a result, additional efforts to support mothers working longer hours due to socioeconomic disadvantage is essential to nurture secure infant attachment, as well as healthy mental and physical child development.

Attenuating the highest proportion of the association between MA and MRBs, our study further highlights the influence of maternal depression on child health. Previous studies suggest that maternal depression may affect how mothers behave and interact with infants, thereby influencing MA. A meta-analysis by Barnes and Theule (2019) using 42 eligible studies found a small but significant association between maternal depression and attachment insecurities overall [16]. Findings showed 20% more cases of lower attachment among infants with depressed mothers compared to mothers without depressive symptoms. Decreased affection and sensitivities demonstrated by depressed mothers is a potential explanation, as depression is characterised by desolate, irritable or vacant moods [48]. Flouri and Ioakeimidi (2018) using the MCS also found that children who experienced maternal depression at 3, 5, 7 and 11 years reported more risky behaviours at 11 years old than children with non-depressed mothers, which was consistent with previous studies [17, 49, 50]. This study consequently underscores the importance of maternal depression as an early-life risk factor mediating an increased risk of MRB uptake through lower MA.

### Strengths and limitations

To our knowledge, this is the first longitudinal study to test whether MA formed during infancy is associated with subsequent risky health behaviours during adolescence in the UK, whilst exploring a wide range of potential confounders and mediators. The significant strengths of our study include the use of data from a large, UK-representative cohort, with longitudinal follow-up from birth to adolescence.

However, as with all observational studies, attrition may be a potential source of bias. The MCS at baseline had a representative sample of 18,296 singleton children born in the UK between September 2000 and January 2002. Yet, by age 17 years, 10,496 children had participated in all subsequent waves. To minimise attrition bias, we used response weights to account for the loss of respondents up to age 17 years, yielding a 74% response rate [23, 51]. The attrition weights adjust the sample composition by taking into account the selective loss of respondents, yet some population groups may still be overlooked. For example, low-income families may be less likely to remain in the cohort or attrition due to MA may underrepresent mothers and infants with severe attachment complications. More information on the MCS attrition weights is available in Ketende and Jones’ (2011) “User Guide to Analysing MCS Data using Stata” [51]. Yet, comparisons between omitted and analytical sample characteristics were similar (Appendix Table 1), indicating no relationship between the missing and observed data.

Combining risky behaviours into a sum score increased statistical power to examine similar explanatory mechanisms together, but the inquiry into links specific to each risky behaviour is complicated. Bivariate regression results presented in Appendix Table 5 investigating associations between individual risky behaviours showed increased odds of most risky behaviours occurring together. However, following anomalous reduced odds in alcohol consumption with gambling and vaping, we also conducted sensitivity analyses to examine any differences without alcohol from the main results. These findings were consistent with the associations observed by the main models and found that maternal depression attenuated the highest proportion of baseline odds (25%) following full adjustment (29%) (Appendix Table 6).

To identify any reporting bias of substance use reported by adolescents, a question on a fictional drug, “Semeron”, was administered in the illegal drug questions [52]. Four cohort members responded “yes” to Semeron attempts alongside illegal drug use. These members were a small proportion (0.04%) of the total final sample (*n* = 7796 after exclusion) and were subsequently removed from the analyses to maintain response validity (Fig. 1).

### Policy and intervention implications

There is an opportunity to integrate and deliver both child and maternal health needs under the Global Strategy for Women’s, Children’s and Adolescent’s Health currently implemented by the World Health Organisation and Every Woman Every Child movement [53]. Aligned with the Sustainable Development Goals, an ongoing target is to reduce premature deaths due to non-communicable diseases by a third and promote mental health by 2030. Present recommended evidence-based intervention postnatal packages include early breastfeeding initiation, responsive caregiving and post-partum maternal depression screening, while adolescent health focuses on psychosocial support and promoting healthy behaviour. Regularly monitoring and supporting these needs throughout the life course by recognising early-life MA as an overarching child development need could innovate health delivery through integrated MRB interventions during adolescence.

A systematic review by MacArthur et al. (2018) highlighted that current adolescent interventions targetting MRBs yield mixed success, as school-based programs were more effective than individual- and family-based agendas [54]. Universal school programmes targeting a larger population of adolescents effectively reduced smoking (OR: 0.77, 95% CI: 0.60–0.97) alcohol consumption (OR: 0.72, 95% CI: 0.56–0.92), illegal drug use (OR: 0.74, 95% C: 0.55–1.00) and antisocial behaviour (OR: 0.81, 95% CI: 0.66–0.98), but not other MRBs [54]. By contrast, despite most family-based interventions focusing on improving parent-child relationships to reinforce family attachment, studies were low to moderate quality, and findings yielded no or little effect on MRBs. These findings suggest a need for better quality family intervention studies to inform policies on early-life MA and adolescent MRBs [54]. In low- and middle-income countries, Gilmore and McAuliffe (2013) also note the implementation of community health workers as an essential cost-effective resource providing maternal and child support promoting secure MA and aiding the recovery of maternal depression in intervention groups [55]. Increasing awareness and guidance from healthcare workers on the shared influence of early-life factors, including socioeconomic, mental and psychosocial influences on mothers and children, could optimise current patient-centred strategies reducing MRBs.

## Conclusions

Preventable risky health behaviours are typically adopted during adolescence and can lead to progressively poor health outcomes. However, intervening within the early stages of child development and addressing wider health determinants has lasting implications on later health trajectories. We show that early-life MA and maternal depression in infancy are potential areas for policy and interventions to prevent MRB uptake in adolescence.

## Supplementary Information

**Additional file 1.**

## Data Availability

Millennium Cohort Study datasets used in this study are available on the UK Data Service website (https://beta.ukdataservice.ac.uk/datacatalogue/series/series?id=2000031) [20]. The data identifier code is SN 2000031.

## Abbreviations

95% CI: 95% Confidence Intervals
A-levels: Advanced levels
GCSE: General Certificate of Secondary Education
MA: Maternal Attachment
MCS: Millennium Cohort Study
MRBs: Multiple Risky Behaviours
NHS MREC: National Health Service Multi-Centre Research Ethics Committee
NVQ: National Vocational Qualification
OECD: Organisation for Economic Cooperation and Development
OR: Odds Ratio
SECs: Socioeconomic Circumstances
UK: United Kingdom
