# Supplementary material for: Early-life maternal attachment and risky health behaviours in adolescence: findings from the United Kingdom Millennium Cohort Study
Source: BMC Public Health. 2021 Nov 8;21:2039. doi: 10.1186/s12889-021-12141-5 (PMC8577004; doi:10.1186/s12889-021-12141-5)
Supplement: Supplementary file 1 — Additional file 1. [file 12889_2021_12141_MOESM1_ESM.docx]

**Additional file 1 – List of Appendices**

- Appendix Figure 1 – Logic model conceptualising the direct and indirect pathways between maternal attachment and multiple risky behaviours
- Appendix Table 1 – Cohort member characteristics by maternal attachment and multiple risky behaviours in omitted and analytical samples.
- Appendix Table 2 – Maternal attachment questions and responses using 6 items selected from the Maternal Postnatal Attachment Scale.
- Appendix Table 3 – Maternal attachment as a continuous variable and its associations with potential explanatory variables
- Appendix Table 4 – Risky behaviours questionnaire items and responses in the Millennium Cohort Study at 17 years
- Appendix Table 5 – Bivariate regressions between risky behaviours at 17 years between maternal attachment and multiple risky behaviours
- Appendix Table 6 – Multiple risky behaviours odds ratios without alcohol consumption (sensitivity analysis) among cohort members of mothers with lower attachment scores.

**Appendix Figure 1**

Logic model conceptualising the direct and indirect pathways between maternal attachment at 9 months and multiple risky behaviours at 17 years (bold arrow line).


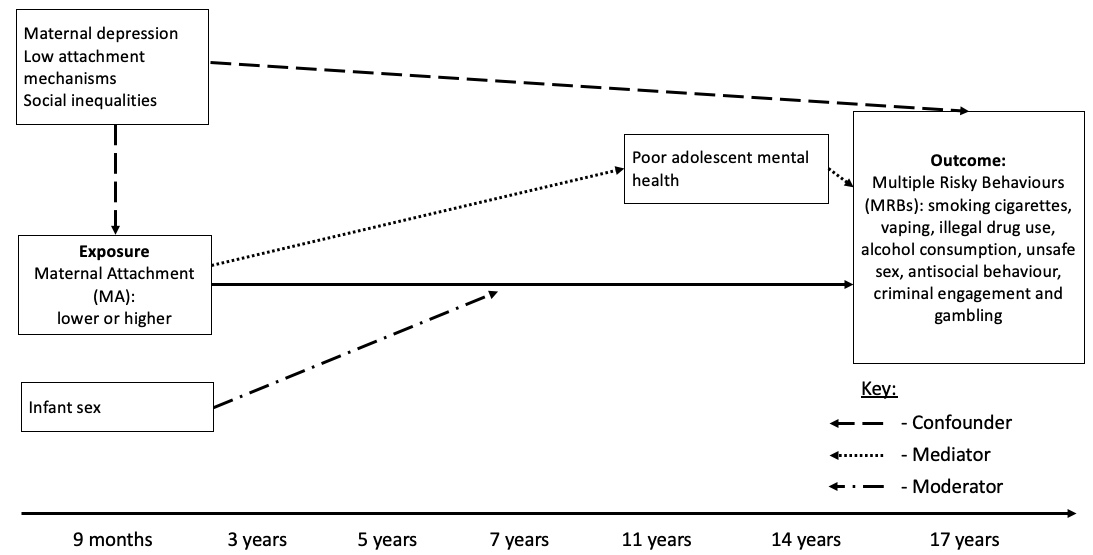


Alongside the direct pathway between the exposure and outcome variables, indirect pathways across the child’s life course may also confound (dashed line), mediate (dotted lines) or moderate (dotted and dashed lines) causal inference. Low attachment mechanisms included multiple births, unplanned pregnancy, infant prematurity, breastfeeding, maternal age at birth. Social inequalities included single-parent status, socioeconomic circumstance (SEC) by maternal education, household income by UK quintiles.

**Appendix Table 1**

Cohort member characteristics by maternal attachment and Multiple Risky Behaviours (MRBs) at 17 years by number of observations (n) and column percentages (%) according to omitted (*n* = 2,700) and analytical (*n* = 7,796) samples.

| Cohort member characteristics | Maternal attachment | | | | | | | | | | | |
| --- | --- | --- | --- | --- | --- | --- | --- | --- | --- | --- | --- | --- |
|  | Omitted from sample | | | | | | Analytical sample | | | | | |
|  | Higher | | Lower | | Total | | Higher | | Lower | | Total | |
|  | n | % | n | % | n | % | n | % | n | % | n | % |
| MRBs at 17 | 214 | 58.7 | 36 | 63.1 | 250 | 59.2 | 4611 | 68.1 | 597 | 72.4 | 5208 | 68.6 |
| Regular smoking | 60 | 24.4 | 15 | 27.3 | 75 | 24.7 | 1371 | 21.8 | 186 | 23.6 | 1557 | 22.0 |
| Regular vaping | 31 | 11.8 | 10 | 20.9 | 41 | 13.0 | 784 | 11.7 | 110 | 12.7 | 894 | 11.8 |
| Alcohol consumption and binge drinking |  |  |  |  |  |  |  |  |  |  |  |  |
| Yes – but never binged | 107 | 28.8 | 21 | 35.6 | 128 | 29.7 | 1881 | 28.5 | 234 | 26.6 | 2115 | 28.4 |
| Yes – and have binged before | 125 | 40.7 | 19 | 39.0 | 144 | 40.4 | 3754 | 58.5 | 467 | 58.6 | 4221 | 58.4 |
| Illegal drugs | 76 | 28.3 | 14 | 31.4 | 90 | 28.7 | 2004 | 31.1 | 302 | 37.6 | 2306 | 31.9 |
| Gambling | 32 | 10.2 | 5 | 21.2 | 37 | 11.7 | 691 | 14.0 | 85 | 11.7 | 777 | 13.7 |
| Criminal engagement | 45 | 22.9 | 10 | 27.5 | 55 | 23.5 | 937 | 18.0 | 136 | 18.8 | 1073 | 18.1 |
| Antisocial behaviour | 114 | 31.3 | 18 | 37.6 | 132 | 32.1 | 2129 | 31.0 | 283 | 35.7 | 2412 | 31.6 |
| Unsafe sex | 101 | 32.2 | 17 | 39.6 | 118 | 33.0 | 2589 | 40.0 | 328 | 42.1 | 2917 | 40.3 |

**Appendix Table 2**

| **Questionnaire**  **item** | **Responses** |
| --- | --- |
| Annoyance with your infant | Almost all the time; very frequently; occasionally; very rarely; never or can’t say |
| Thoughts of your infant* | Never; very rarely; occasionally; very frequently; almost all the time or can’t say |
| Leaving your infant* | I always feel rather relieved; I often feel rather relieved; I have mixed feelings of both sadness and relief; I often feel rather sad; I always feel rather sad or can’t say |
| Competence as a mother | Very incompetent and lacking in confidence; fairly incompetent and lacking in confidence; fairly competent and confident, very competent and confident or can’t say |
| Patience with your infant | I am very impatient; I am a bit impatient; I am extremely patient or can’t say |
| Resentment with your infant | I find that I resent it quite a lot; I find that I resent it a fair amount; I find that I resent it a bit; I don’t resent it at all or can’t say |

Maternal attachment questions and responses using 6 items selected from the Condon (1998) Maternal Postnatal Attachment Scale.

**Footnote:** Thoughts of your infant and leaving your infant questionnaire items’ responses were reversed (marked by asterisks, *). “Can’t say” responses were recoded to missing (.).

**Appendix Table 3**

Characteristics of the complete case UK Millennium Cohort Study (MCS) population by higher and lower maternal attachment (MA) at 9 months as a continuous variable (*n* = 7,796).

| Potential explanatory block model | Variable | Association with MA as a continuous variable (P-value) |
| --- | --- | --- |
| Baseline | Multiple Risky Behaviours (MRBs) | 0.07 |
| 1-4 | Infant sex | <0.001 |
| 1 | Multiple births | 0.78 |
|  | Unplanned pregnancy | 0.80 |
|  | Infant prematurity | 0.91 |
|  | Breastfeeding | 0.13 |
|  | Maternal age at birth | <0.001 |
| 2 | Maternal depression | <0.001 |
| 3 | Poor adolescent mental health | 0.84 |
| 4 | Single-parent status | <0.001 |
|  | Socioeconomic circumstance by maternal education | <0.001 |
|  | Household income by UK quintiles | <0.001 |

**Appendix Table 4**

Risky behaviours questionnaire items and responses in the Millennium Cohort Study at 17 years.

| **Risky Behaviours Measures** | **Questionnaire items** | **Responses** | |
| --- | --- | --- | --- |
| Substance use | Ever tried: smoking (cigarettes or e-cigarettes), alcoholic drinks or illegal drugs (marijuana, cocaine, acid/LSD, ecstasy, ketamine, mephedrone, psychoactive substances, speed/amphetamine and “Semeron” use)? | Smoking (cigarettes or e-cigarettes) | Never tried smoking, tried once, sometimes but never smoke now, sometimes but not as often as once a week, between 1 and 6 times a week, more than 6 times a week |
|  |  | Alcoholic drinks | Never, 1-2 times, 3-5 times, 6-9 times, 10-19 times, 20-39 times, or 40 or more times |
|  |  | Illegal drugs | Yes or no |
| Gambling | With your own money, have you: used fruit machines, placed a private bet for money (e.g. with friends), placed a bet at a betting shop (e.g. on football or horseracing), or any other gambling? | Yes or no | |
| Unsafe sex | Had sexual intercourse without contraception, tested for STIs, pregnancies or received a positive diagnosis following an STI test? | Yes or no | |
| Antisocial behaviour | Ever: noisy or rude in a public place (public nuisance), graffitied, vandalised, shoplifted, stolen from someone (e.g. mobile phone, money), burgled someone’s home, assaulted (shoved, hit, slapped, punched someone), assaulted someone with a weapon, carried a knife or weapon, part of a gang, hacked or sent a virus (cybercrime), stolen a vehicle, deliberately set something on fire that should not have, harassed someone (including sexual harassment), used someone else’s credit card, sent pictures or spread rumours about someone via email or phone? | Yes or no | |
| Criminal engagement | Ever been: stopped and questioned, cautioned or warned, or arrested by the police? | Yes or no | |

**Footnote:** All risky behaviour responses were limited to activity within the last 12 months. For unsafe sex questions, whether cohort members have had sex was used as a reference category.

**Appendix Table 5**

Associations between individual risky behaviours at 17 years using bivariate logistic regressions.

| Risky behaviour variable | Engagement in other risky behaviours at 17 | | | | | | | | |
| --- | --- | --- | --- | --- | --- | --- | --- | --- | --- |
|  | Regular smoking | Regular vaping | Alcohol consumption / binge drinking | Illegal drugs | Gambling | Unsafe sex | Criminal engagement | | Antisocial behaviour |
|  | ^b^ % (95% CI) | % (95% CI) | % (95% CI) | % (95% CI) | % (95% CI) | % (95% CI) | % (95% CI) | % (95% CI) | |
| Regular smoking | – | 69.0 (65.10 – 72.69) | 7.5 (6.33 – 8.86) / 32.6 (30.78 – 34.47) | 50.7 (48.24 – 53.20) | 28.1 (23.96 – 32.59) | 36.5 (34.22 – 38.82) | 41.0 (37.34 – 44.72) | 35.8 (33.52 – 38.12) | |
| ^a^ Odds Ratio (OR) | – | 12.70 (10.55– 15.28 ** | 2.70 (1.80 – 4.04) ** / 16.09 (10.89 – 23.76) ** | 13.22 (11.20 – 15.61) ** | 2.04 (1.66 – 2.51) ** | 4.46 (3.90 – 5.10) ** | 4.81 (4.03 – 5.74) ** | 3.26 (2.83 – 3.76) ** | |
| Regular vaping | 38.2 (35.37 – 41.15) | – | 3.4(2.60 – 4.58) / 17.8 (16.40 – 19.29) | 26.6 (24.44 – 28.85) | 19.4 (16.08 – 23.17) | 20.2 (18.20 – 22.27) | 20.3 (18.55 – 22.25) | 20.7 (18.67 – 22.96) | |
| OR | 12.70 (10.55 – 15.28 ** | – | **0.87 (0.57 – 1.33)** / 5.27 (3.88 – 7.14) ** | 7.32 (6.15 – 8.70) ** | 2.75 (2.16 – 3.50) ** | 3.85 (3.16 – 4.68) ** | 4.45 (3.54 – 5.59) ** | 3.15 (2.65 – 3.74) ** | |
| Alcohol consumption / binge drinking |  |  |  |  |  |  |  |  | |
| Yes – but never binged | 10.0 (8.4 – 11.7) | 8.3 (6.30 – 10.78) | – | 10.9 (9.48 – 12.49) | 19.1 (15.95 – 22.69) | 19.3 (17.59 – 21.22) | 19.3 (16.46 – 22.49) | 18.2 (16.35 – 20.14) | |
| Yes – have binged before | 88.0 (86.25 – 89.83) | 87.1 (84.34 – 89.43) | – | 87.0 (85.10 – 88.60) | 70.8 (66.12 – 75.07) | 77.8 (75.74 – 79.62) | 75.0 (71.74 – 78.01) | 73.2 (70.66 – 75.66) | |
| OR | 2.70 (1.80 – 4.04) ** / 16.09 (10.89 – 23.76) ** | **0.87 (0.57 – 1.33**) / 5.27 (3.88 – 7.14) ** | – | 2.69 (1.93 – 3.75) ** / 17.99 (12.96 – 24.97) ** | **0.86 (0.61 – 1.22) /** 1.92 (1.39 – 2.65) ** | 4.13 (3.03 – 5.62) ** / 12.61 (9.35 – 17.00) ** | 1.64 (1.18 – 2.28) **/ 4.06 (3.10 – 5.31) ** | 1.04 (0.87 – 1.27) / 2.76 (2.32 – 3.27) ** | |
| Illegal drugs | 77.1 (74.17 – 79.78) | 72.9 (69.35 – 76.28) | 12.4 (10.68 – 14.44) / 48.7 (46.60– 50.82) | – | 44.3 (39.80 – 48.87) | 51.3 (48.81 – 53.68) | 57.9 (54.49 – 61.23) | 52.1 (49.32– 54.85) | |
| OR | 13.22 (11.20 – 15.61) ** | 7.32 (6.15 – 8.70) ** | 2.69 (1.93 – 3.75) ** / 17.99 (12.96 – 24.97) ** | – | 2.10 (1.75 – 2.53) ** | 4.19 (3.68 – 4.78) ** | 4.45 (3.80 – 5.22) ** | 3.61 (3.15 – 4.13) ** | |
| Gambling | 21.6 (18.72 – 24.80) | 27.6 (23.50 32.03) | 8.6 (7.18 – 10.23) / 17.3 (15.84– 18.87) | 20.2 (18.13 – 22.52) | – | 17.5 (15.77 – 19.32) | 25.0 (22.42 – 27.82) | 22.1 (19.78 – 24.56) | |
| OR | 2.04 (1.66 – 2.51) ** | 2.75 (2.16 – 3.50) ** | **0.86 (0.61 – 1.22)** / 1.92 (1.39 – 2.65) ** | 2.10 (1.75 – 2.53) ** | – | 1.66 (1.39 – 1.98) ** | 2.66 (2.20 – 3.22) ** | 2.53 (2.11 – 3.05) ** | |
| Unsafe sex | 67.9 (65.01 – 70.64) | 68.6 (64.63 – 72.41) | 27.3 (24.89 – 29.94) / 53.6 (51.56 – 55.68) | 62.8 (60.07 – 65.47) | 46.8 (42.59– 51.15) | – | 58.5 (54.87 – 62.12) | 51.2 (48.62 – 53.84) | |
| OR | 4.46 (3.90 – 5.10) ** | 3.85 (3.16 – 4.68) ** | 4.13 (3.03 – 5.62) ** / 12.61 (9.35 – 17.00) ** | 4.19 (3.68 – 4.78) ** | 1.66 (1.39 – 1.98) ** | – | 3.07 (2.60 – 3.63) ** | 2.00 (1.79 – 2.23) ** | |
| Criminal engagement | 41.5 (37.72 – 45.35) | 44.3 (38.96 – 49.70) | 11.4 (9.80 – 13.33) / 24.2 (22.25 – 26.29) | 34.9 (31.96 – 37.90) | 33.5 (29.37 – 37.79) | 28.8 (26.49 – 31.24) | – | 33.9  (31.01 – 36.91) | |
| OR | 4.81 (4.03 – 5.74) ** | 4.45 (3.54 – 5.59) ** | 1.64 (1.18 – 2.28) **/ 4.06 (3.10 – 5.31) ** | 4.45 (3.80 – 5.22) ** | 2.66 (2.20 – 3.22) ** | 3.07 (2.60 – 3.63) ** | – | 4.04 (3.45 – 4.74) ** | |
| Antisocial behaviour | 53.3 (50.19 – 56.34) | 55.7 (51.95 – 59.39) | 20.3 (18.11 – 22.73) / 40.19 (38.09 – 42.32) | 51.0 (48.47 – 53.57) | 48.1 (44.21 – 52.08) | 41.0 (39.03 – 43.92) | 56.0 (52.53 – 59.43) | – | |
| OR | 3.26 (2.83 – 3.76) ** | 3.15 (2.65 – 3.74) ** | 1.04 (0.87 – 1.27) / 2.76 (2.32– 3.27) ** | 3.61 (3.15 – 4.13) ** | 2.53 (2.11 – 3.05) ** | 2.00 (1.79 – 2.23) ** | 4.04 (3.45 – 4.74) ** | – | |

**Footnote:** * = p<.05, ** = p<.001. a = Odds Ratio, b % = column percentage. Results in bold indicate reduced odds associated with alcohol consumption. Total number of participants = 7,796.

**Appendix Table 6**

Multiple risky behaviours odds ratios (OR) without alcohol consumption (sensitivity analysis) at 17 years among cohort members from mothers with lower attachment identified at 9 months (*n* = 7,796).

| Model | Adolescent multiple risky behaviours by lower maternal attachment | | Proportion attenuated (%) |
| --- | --- | --- | --- |
|  | OR | 95% CI |  |
| Baseline | 1.24* | 1.01 – 1.53* | Reference |
| 1: Low attachment mechanisms | 1.21 | 0.98 – 1.50 | 13% |
| 2: Maternal depression | 1.18 | 0.96 – 1.46 | 25% |
| 3: Poor adolescent mental health | 1.21 | 0.98 – 1.48 | 13% |
| 4: Social inequalities | 1.21 | 0.98 – 1.48 | 13% |
| 5: Fully adjusted | 1.17 | 0.96 – 1.44 | 29% |

**Footnote:** OR = Odds Ratio, 95% CI = 95% Confidence Intervals. Baseline model= unadjusted univariate analysis between MA (Maternal Attachment) and MRBs (Multiple Risky Behaviours); Model 1 (low attachment mechanisms) = adjusted for multiple births + unplanned pregnancy + infant prematurity + breastfeeding + maternal age at birth + infant sex; Model 2 (maternal depression) = adjusted for maternal depression + infant sex; Model 3 (poor adolescent mental health) = adjusted for poor adolescent mental health + infant sex; Model 4 (social inequalities) = adjusted for single parent status + socioeconomic circumstance + household income + infant sex and Model 5 was fully adjusted for all risk factors. Proportion attenuated was calculated by the formula: (100 x (Baseline model OR – Adjusted OR) / (Baseline model OR – 1)). Total number of complete case sample participants (n) = 7,796.
